# Post-keratoplasty Infectious Keratitis: Epidemiology, Risk Factors, Management, and Outcomes

**DOI:** 10.3389/fmed.2021.707242

**Published:** 2021-07-07

**Authors:** Anna Song, Rashmi Deshmukh, Haotian Lin, Marcus Ang, Jodhbir S. Mehta, James Chodosh, Dalia G. Said, Harminder S. Dua, Darren S. J. Ting

**Affiliations:** ^1^Newcastle University, Newcastle upon Tyne, United Kingdom; ^2^Department of Ophthalmology, Cambridge University Hospitals National Health Service Foundation Trust, Cambridge, United Kingdom; ^3^State Key Laboratory of Ophthalmology, Zhongshan Ophthalmic Center, Sun Yat-sen University, Guangzhou, China; ^4^Singapore National Eye Centre, Singapore Eye Research Institute, Singapore, Singapore; ^5^Harvard Medical School, Massachusetts Eye and Ear, Boston, MA, United States; ^6^Academic Ophthalmology, Division of Clinical Neuroscience, School of Medicine, University of Nottingham, Nottingham, United Kingdom; ^7^Department of Ophthalmology, Queen's Medical Centre, Nottingham, United Kingdom

**Keywords:** corneal graft, corneal infection, corneal transplant, corneal ulcer, eye bank, interface infectious keratitis, keratoplasty, steroid

## Abstract

Post-keratoplasty infectious keratitis (PKIK) represents a unique clinical entity that often poses significant diagnostic and therapeutic challenges. It carries a high risk of serious complications such as graft rejection and failure, and less commonly endophthalmitis. Topical corticosteroids are often required to reduce the risk of graft rejection but their use in PKIK may act as a double-edged sword, particularly in fungal infection. The increased uptake in lamellar keratoplasty in the recent years has also led to complications such as graft-host interface infectious keratitis (IIK), which is particularly difficult to manage. The reported incidence of PKIK differs considerably across different countries, with a higher incidence observed in developing countries (9.2–11.9%) than developed countries (0.02–7.9%). Common risk factors for PKIK include the use of topical corticosteroids, suture-related problems, ocular surface diseases and previous corneal infection. PKIK after penetrating keratoplasty or (deep) anterior lamellar keratoplasty is most commonly caused by ocular surface commensals, particularly Gramme-positive bacteria, whereas PKIK after endothelial keratoplasty is usually caused by *Candida spp*. Empirical broad-spectrum antimicrobial treatment is the mainstay of treatment for both PKIK, though surgical interventions are required in medically refractory cases (during the acute phase) and those affected by visually significant scarring (during the late phase). In this paper, we aim to provide a comprehensive overview on PKIK, encompassing the epidemiology, risk factors, causes, management and outcomes, and to propose a treatment algorithm for systematically managing this challenging condition.

## Introduction

Corneal opacity is the 5th leading cause of blindness globally, with around 6 million of the population being affected (1–3). Among all aetiologies, infectious keratitis (IK), also known as microbial keratitis or infectious corneal ulceration, consistently features as the most common culprit of corneal blindness, particularly in the developing countries. IK is a painful and potentially blinding condition that may require hospital admission for intensive medical treatment and/or surgical interventions (1, 2). It can be caused by a wide array of organisms, including bacteria, fungi, viruses, parasites or mixed infection (1, 4, 5). Nonetheless, as the ocular surface is equipped with a multifaceted defence system (6, 7), IK rarely occurs in the absence of any predisposing factor. Commonly reported risk factors include contact lens wear, trauma, ocular surface disease and ocular surgery, particularly keratoplasty (1, 2, 8, 9).

Post-keratoplasty infectious keratitis (PKIK) represents a challenging clinical entity that often poses significant diagnostic and therapeutic challenges. It is uniquely different from a “standard” IK in several ways. Firstly, the occurrence of IK in a graft can result in potentially devastating complications such as graft rejection, failure and endophthalmitis (10–12). Secondly, topical corticosteroids are often required to reduce the risk of graft rejection. However, in the event of PKIK, the use of topical corticosteroids may act as a double-edged sword as it can worsen the infection during the acute phase, particularly in fungal infection. Furthermore, there has been a paradigm shift in keratoplasty in the past decade where lamellar keratoplasty such as deep anterior lamellar keratoplasty (DALK), Descemet stripping automated endothelial keratoplasty (DSAEK), pre-Descemet's endothelial keratoplasty (PDEK), and Descemet membrane endothelial keratoplasty (DMEK) have superseded penetrating keratoplasty (PKP) as the preferred choice of keratoplasty for anterior and posterior corneal pathologies (13–19). However, this has also resulted in a number of complications that are not usually observed following PKP, including graft detachment and interface infectious keratitis (IIK) (20–23). IIK, a unique subtype of PKIK which can develop after ALK or EK, is a difficult-to-treat condition as the sequestration of the infective microorganisms at the graft-host interface hinders access for obtaining samples for microbiological culture and for topical antimicrobial treatment to effectively reach and treat the affected site.

As corneal transplant is the most commonly performed type of transplant worldwide, the occurrence of PKIK and its resultant complications have a significant impact. In this paper, we aim to provide a comprehensive overview on PKIK, encompassing the epidemiology, risk factors, causes, management and outcomes, and to propose a treatment algorithm for systematically managing this challenging condition.

## Method of Literature Search

We searched PubMed (January 1980–2021) for relevant articles related to IK after keratoplasty. Keywords such as “infectious keratitis,” “corneal ulcer,” “corneal infection,n, “microbial keratitis,” “keratoplasty,” “corneal transplantation,” and “corneal graft” were used. Only articles published in English were included for the review. The search was first performed on 10 September 2020 and was last updated on 05 January 2021. The literature search retrieved 328 articles, of which the abstracts and titles were screened for those that fulfilled the eligibility criteria. After excluding ineligible studies, 48 were included in the qualitative synthesis. A PRISMA flow chart is provided in Supplementary Figure 1. The demographic factors, clinical characteristics and outcomes of PKIK of large case series (>500 cases) are summarised in Table 1.

Table 1Summary of post-keratoplasty infectious keratitis (PKIK) based on large case studies (>500 cases), in the order of chronology.

**Table:** 

**Authors**	**Year**	**Study period**	**Region**	**No. of grafts**	**Types of graft**	**No. of eyes with PKIK**	**Age, years (mean ± SD)**	**Female %**	**Incidence %**	**Time of PKIK after keratoplasty (months)**
Dohse et al. (24)	2020	2007–2018	US	2,098	PK and EK	86	64.7 ± 21.7	59.3	4.1 (PK: 5.9, EK: 1.3)	28.7 (28.5 for PK, 30.4 for EK)
Griffin et al. (25)	2020	2004–2015	UK	1,508	PK, DALK, and epikeratophakia	66 (72 episodes)	56.0 ± 20.7	49	4.77	25 months
Okonkwo et al. (26)	2018	1997–2014	UK	759	PK, DALK, and DSAEK	41 (59 episodes)	73.0 ± 19.4	53.7	5.4	–
Sun et al. (27)	2017	2000–2009	Taiwan	871	PK	52 (67 episodes)	65.5 ± 16.9	–	7.7	27.1 ± 28.0 days (range, 0–86 days)
Chen et al. (28)	2017	2003–2007	Taiwan	648	PK	42	49.1 ± 21.5	40.5	6.5	12.0 ± 9.5 months
Edelstein et al. (29)	2016	2007–2014	US	354,930	PK, EK, and ALK	66	–	–	0.02	29 days (1–216 days range)
Constantinou et al. (30)	2013	1998–2008	Australia	650	PK	122	75.0 ± 14.8 (failed graft), 61.8 ± 16.3 (clear graft)	58.8	18.8	72.0 ± 32.4 (failed graft), 114.0 ± 97.2 (clear graft)
Wagoner et al. (12)	2007	1998–2002	US	2,103	PK	102	50.4	42.2	4.9	38.2% occurred within 12 months
Tavakkoli and Sugar (31)	1994	1976–1992	US	885	PK	36	–	–	4.9	–
Leahey et al. (32)	1993	1976–1992	US	773	PK	18	58.9	72.2	–	21.5 months (range 1–53 months)
Bates et al. (33)	1990	1983–1988	UK	1,700	PK	30	55	41	1.76	10 months (range 1–168)
Fong et al. (34)	1988	1978–1985	US	2,006	PK	66 (68 episodes)	61	–	3.3	–
Al-Hazzaa and Tabbara (35)	1988	1983–1986	Saudi Arabia	947	PK	113	–	31	11.9	5.4 months (range 10 days−12 months)

**Table:** 

**Authors**	**Risk factors (%)**	**Organisms (%)**					**Complications (%)**	**Clear graft (%)**	**Visual outcome (logMAR)**	**Mean follow-up duration (months)**
		**GP**	**GN**	**F**	**V**	**P**				
Dohse et al. (24)	TS (82.6), GF (6.2), corneal scar (5.6)	PK: 44 EK: 45.4	PK: 21.3 EK: 18.2	PK: 10.7 EK: 9.1	–	–	GF (67.4), repeat transplantation or keratoprosthesis (33.7), enucleation or evisceration (5.8)	32.1	8.1% (0.0–0.3); 11.6% 0.4–0.6; 30.2% (0.7–1.3); 43.8% (counting fingers or worse)	47.8 (PK), 38.6 (EK)
Griffin et al. (25)	TS (89), TG (32), SR (26), HSV (25), atopy/eczema (22), GF (18)	73	23	4	–	–	GF (11), graft rejection episode (3), perforation (13), crystalline keratopathy (6), orbital cellulitis (1), endophthalmitis (1), further PK (24), evisceration (4)	–		
Okonkwo et al. (26)	GF (61.4), TG (59.6), SR (19.3)	30.5	18.6	8.5	–	–	Corneal scarring (39), GF (7.3), PED (39), corneal neovascularisation (15), graft rejection (7.3), corneal perforation (4.9)	60		
Sun et al. (27)	TG, SR, regraft (8.3), corneal scar (7.6), bullous keratopathy (5.8)	57.9	22.4	19.7	–	–	Therapeutic PK, evisceration	65.7	–	37.0
Chen et al. (28)	SR (31), lid abnormalities (23.8), PED (23.8), CL (14.3), dry eye (11.9), prior ejection episodes (4.8)	Y	Y	Y	–	–	GF (71.4), hypopyon (21.4), corneal perforation (14.3), wound dehiscence (11.9), endophthalmitis (4.8)	85	33.3% VA >1, 66.7% VA <1	
Edelstein et al. (29)	–	5	7	81	7		GF, endophthalmitis	–		
Constantinou et al. (30)	TS (88.2), TG (50.9), ocular surface disease (19.6), PED (9.8), CL (2.0)	56.9	18.6	1.7	10.2	–	GF (51)	49	1.8 ±1.0 in clear-graft group; 1.7 ± 0.9 in failed graft group	
Wagoner et al. (12)	TS (73.5), SR (71.6), TG (38.2), previous infection (18.6), previous rejection (13.7)	82.8	16.5	–	–	–	GF (46)	—-	7.8% (≥0.3); 20.6% (>1.0)	32.4
Tavakkoli and Sugar (31)	PED (64), SR (36)	–	–	–	–	–		50		
Leahey et al. (32)	TS (72.2)	94.4	22.2	–	–	–	Scarred corneas (17), GF (16), endophthalmitis	67		
Bates et al. (33)	TS (96.7), SR (33.3), TG (33.3), GF or recent rejection (23.3), systemic atopy (20), PED (10), CL (3.3)	Y	Y	Y	–	Y	GF (13), corneal perforation (17), endophthalmitis (13), regraft (53)	23		
Fong et al. (34)	TS (85), SR (50), CL (26), TG (19), previous HSV (15), GF (15), PED (15)	59	38	6	–	–	Descemetocele (6), corneal perforation (12), endophthalmitis (6), enucleation/evisceration (9), wound dehiscence (24), graft failure (16), emergency repeat PK (19), elective repeat PK (13)	–	10% (no light perception)	
Al-Hazzaa and Tabbara (35)	Trichiasis (39), PED (38), SR (33), CL (30), dry eye syndrome (27)	Y	Y	0.1	0.3	–	Endophthalmitis (4)	–	24% (1.3 or better); 72% (counting fingers to light perception)	>6 months post-operatively

*PK, Penetrating keratoplasty; EK, Endothelial keratoplasty; DALK, Deep anterior lamellar keratoplasty; CL, Contact lens; SR, Suture-related problems; GF, Graft failure; PED, Persistent epithelial defect; HSV, Herpes simplex virus; TS, Topical steroids; TG, Glaucoma drops*.

## Epidemiology

### Incidence/Prevalence

The incidence of PKIK differs considerably across different countries, with a higher incidence observed in developing countries than developed countries. Depending on the study design, patient cohort and follow-up duration, the incidence of PKIK in developed countries ranges from 0.02 to 7.9% (Table 1) (26, 28, 29, 31, 33, 36–44). The incidence of PKIK in developing countries is less well documented within the literature, with a higher overall incidence of up to 9.2–11.9% (35, 45). The higher incidence may be attributable to the reduced access to healthcare, poor follow-up compliance, lower level of education, increased risk due to trauma and poor hygiene, poverty and a higher proportion of primary keratoplasty performed as therapeutic keratoplasty for IK (35, 45).

### Age

PKIK affects patients of all age groups, with the majority of cases reported in the literature being between the range of 17–95 years (12, 26, 28, 33, 34, 36, 46–50). This reflects the varied indications for keratoplasty such as keratoconus, pseudophakic bullous keratopathy, Fuchs endothelial dystrophy and corneal ulceration, scarring, or perforation within the adult population (12, 51). In comparison, the main indications for keratoplasty within the paediatric population include keratoconus, regraft, and herpes simplex keratitis (HSK), with a higher preponderance of congenital conditions such as anterior segment dysgenesis (including Peter's anomaly), congenital hereditary endothelial dystrophy and sclerocornea, amongst others (52–54). A study in Denmark evaluated keratoplasties performed in children under 16 over a 40-year period and found that infection was responsible for 20% of failed grafts (55). In a longitudinal retrospective study of 168 paediatric eyes in India, PKIK occurred in 29% of eyes and was responsible for 50% of failed grafts (45). However, the most common indication for keratoplasty in this study was infectious keratitis (43%), which was associated with a high recurrence rate (56).

### Gender

There does not appear to be a gender predilection amongst PKIK. Studies conducted in the Taiwan (51–60%) and Turkey (57%) have found a marginally higher preponderance amongst males (10, 28, 48), whereas a slightly higher preponderance in females was seen in the United States (56–60%) and Korea (57%) (31, 47, 50).

### Socioeconomic Status, Level of Education, and Occupation

Patients from rural regions with lower socioeconomic status and lower levels of education have reduced access to healthcare and are less likely to attend follow-up appointments following keratoplasty. This is reflected within a study conducted in India whereby 75% of patients were from rural communities, with a high rate (28%) of PKIK being observed (45). Additionally, a China study reported farmers to be a significant independent risk factor of PKIK due to higher risk of trauma, particularly from plants resulting in fungal keratitis (57). These populations also have a higher risk for non-compliance of post-operative medication administration and hygiene (58).

### Influence of the Types of Keratoplasty

The types of keratoplasty, including PKP, DALK and EK, have also been shown to greatly influence the incidence, risk and types of PKIK (i.e. ocular surface-related infection or IIK). A large retrospective cohort study of 2,098 keratoplasty performed between 2007 and 2018 in the US observed a PKIK incidence of 5.9% and 1.3% following PKP and EK, respectively (24). The higher proportion of PKIK occurring in PKP (93%) compared to DALK (6%) and EK (0%) was similarly depicted in a UK study of 1,508 grafts (25). The higher proportion of PKIK after PKP hinges on a combination of factors, including the indication for surgery, the use of sutures, and the prolonged use of topical corticosteroids. The indications for EK tend to be non-infective causes such as endothelial dystrophy, whereas a wider range of ocular comorbidities indicated for PKP may include IK and repeat (high-risk) grafts (24). Additionally, the requirement for corneal sutures in PKP, compared to EK, poses substantial risk of IK. This however does not completely explain the difference between PKP and DALK, with both procedures requiring the same number of sutures, though DALK usually does not require long-term topical corticosteroids due to zero-risk of endothelial graft rejection (59).

Conversely, a retrospective study using data from the Eye Bank Association of America analysing all adverse events of corneal grafts found a higher proportion of PKIK in EK (67%) compared to PKP (29%) and ALK (3%) (29). However, it is important to note that this study only included cases of PKIK that were caused by graft-transmitted infection. In addition, when taking into account the total number of each procedure performed (PKP/ALK/EK), the incidence of graft-transmitted infection was similar between EK and ALK (both 2.6 cases per 10,000 grafts) but higher than PKP (0.9 per 10,000 grafts). Interestingly, a higher rate of fungal infection was observed when compared to non-US studies, possibly related to the lack of antifungal agent in the corneal storage medium in the US. (11, 29). The authors also noted a 1.5–3 times higher risk of fungal infection following EK (compared to ALK and PKP), potentially related to the increased warming time associated with the preparation of EK tissues in the eye bank (29).

## Risk Factors

### Topical Corticosteroids

**Figure 1:**
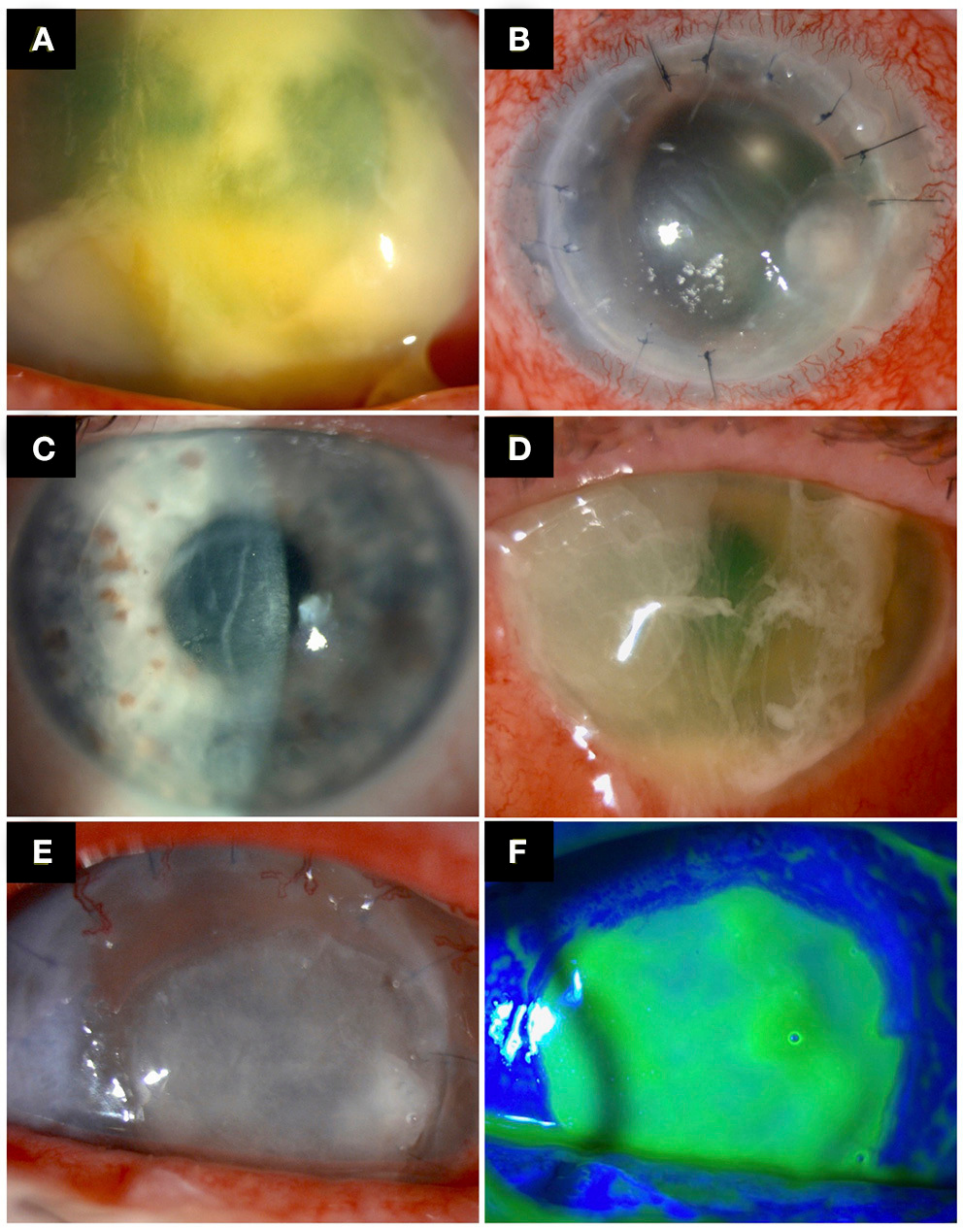
Examples of post-keratoplasty infectious keratitis (PKIK). **(A)** A case of PKIK caused by *Streptococcus pneumonia* in an eye after Descemet membrane endothelial keratoplasty, while on topical corticosteroids. **(B)** A case of suture-related PKIK caused by *Staphylococcus aureus* in an eye after penetrating keratoplasty. **(C,D)** A case of PKIK caused by *Pseudomonas aeruginosa* in an eye with failed Descemet stripping automated endothelial keratoplasty with bullous keratopathy, while on topical steroids. **(C)** demonstrates the presence of decompensated corneal graft prior to the infection. **(E,F)** A case of PKIK caused by *Moraxella catarrhalis* in an eye with failed penetrating keratoplasty with bullous keratopathy, while on topical steroids.

Topical corticosteroids are usually administered following keratoplasty to reduce the risk of graft rejection (60). As such, the majority of studies have found topical corticosteroids to be the main contributing factor (72.2–100%) for PKIK, primarily attributed to its local immunosuppressive effect (12, 25, 32–34, 41, 42, 47, 50) (Figure 1A). A US study observed that 82.6% eyes that developed PKIK were on topical corticosteroid therapy, of which the rates between PKP and EK were comparative at 81.3 and 90.9%, respectively (24). Constantinou et al. (30) performed a retrospective study evaluating non-suture-related PKIK after PKP between 1998 and 2008 in Australia. Long-term topical corticosteroids use was noted in 88% of eyes with PKIK, with 61% eyes developed infection more than 2 years after PKP. Similarly, a UK study (25) observed 89% of their patients developed PKIK (after PKP or DALK) whilst on topical corticosteroids, with a median time of developing IK at 25 months post-keratoplasty (25). This is an interesting observation as one would expect PKIK to develop sooner if the use of topical corticosteroids is directly implicated in the pathogenesis of PKIK since it is often used at a higher frequency and dose during the early postoperative period. Plausible explanations for late occurrence of PKIK include the occurrence of loose or broken sutures, the development or exacerbation of ocular surface diseases such as dry eyes and neurotrophic keratopathy (with persistent epithelial defect), and graft failure with resultant bullous keratopathy (25, 30). In addition, while many studies reported the association of PKIK and use of topical corticosteroids, they did not examine the proportion of grafts that did not develop PKIK while on topical corticosteroids. Future studies examining the incidence of PKIK in all corneal grafts while on topical corticosteroids (including those that did not develop PKIK) would be of clinical interest.

### Suture-Related Problems

Suture-related problems are a major risk factor for IK and has been implicated in 20–50% cases of PKIK, mainly after PKP and DALK (12, 34, 42, 61, 62) (Figure 1B). Furthermore, suture complications increase the risk of graft rejection and failure (42, 63). The occurrence rate of PKIK caused by loose or broken sutures is reported to be as high as 71.6% (10, 12, 25, 26, 28, 36, 39, 43, 49). Four main causes of suture loosening have been described, which include corneal deturgescence, incomplete epithelialisation over the suture material, suture degradation (exacerbated by corneal vascularisation around the sutures), and cheese-wiring (39). The broken or loose suture is implicated in PKIK by causing a resultant epithelial defect that can be contaminated by environmental and ocular surface commensals (39). Cheese-wiring is seen particularly in corneas with keratoconus whereby little support is offered by the thin host cornea (39). In addition, patients with keratoconus are often affected by atopic disease, which increases the postoperative risk of corneal vascularisation around the graft sutures, loose/broken sutures, and graft failure (64). A greater propensity for suture-related infections seems to occur within the interpalpebral zone, likely due to the increased risk of exposure and reduced protection of the eyelids (34, 42).

Suture-related problems generally occur either within 1 year or around 30 months following keratoplasty (37, 39, 42, 63, 65, 66). The reason for the bimodal peak noted in these studies may be due in part to the process of suture-loosening and surgeons' preference as some may remove all corneal graft sutures at 12–18 months post-keratoplasty. Corneal deturgescence and incomplete epithelialization over the suture may result in an earlier onset of infection, whereas suture degradation and cheese-wiring of the corneal tissue contribute to a later onset (39, 42). Christo et al. advocated the removal of sutures as soon as the graft-wound interface is healed at 1 year for vascularised recipients and 18 months for all other cases to minimise the risk of suture-related PKIK, with earlier removal in children (39, 67). However, individualised care is necessary due to variable speed in wound healing (e.g., slower in elderly patients) whereby wound dehiscence or large changes in keratometry can occur upon premature suture-removal (39, 68).

### Previous History of IK

Keratoplasty serves as a useful therapeutic modality in managing patients with IK. It can be performed in the form of optical keratoplasty for visual rehabilitation (by removing the corneal scar) or in the form of therapeutic keratoplasty to manage active, medically refractory IK (15, 69). However, the occurrence of PKIK following therapeutic keratoplasty is high (6–41%) and the risk may be influenced by the type of previous infection (56, 70–72). Wagoner et al. (12) observed 18.6% of those that developed bacterial PKIK were associated with a history of previous bacterial keratitis. Fungal recurrence rates are variable with a range of 7.4–32.7%, with most recurrences presenting within 2 weeks of surgery (73, 74). Due to the propensity of fungi for deep-seated infections with corneal penetration and anterior chamber invasion, the final outcomes of graft clarity (51–84%) and final cure rate requiring no further regrafts (69–90%) are reduced in comparison to a recurrence of bacterial keratitis (69–90% and 90–100%, respectively) (73–76). Therapeutic keratoplasty performed for refractory *Acanthamoeba* keratitis (AK) is often unsuccessful in elimination of the infection, necessitating repeat grafts with resultant guarded outcome (71). In addition, recurrence of HSK post-keratoplasty is common, with ~50% seen within the first 2 years following PKP and in 33% at 3 years for DALK, and is usually associated with a high risk of graft rejection and failure (77–79). However, it is noteworthy to mention that most of these studies were conducted more than 1–2 decades ago. Recognition of the high recurrent risk of HSK had led to increased use of prophylactic oral aciclovir post-keratoplasty, which could reduce the risk of HSK recurrence and resultant graft rejection/failure (80).

### Ocular Surface Diseases

Ocular surface diseases constitute a significant risk factor for PKIK (following PKP and DALK) due to the poor ocular environment, breakdown of corneal epithelium, and reduced tear film quantity and quality (including its antimicrobial compounds) (81). Causes include dry eye disease (22.2–28.2%), blepharitis (23.8–43.6%), persistent epithelial defect or neurotrophic keratopathy (14.3–77.8%), trichiasis (13.3–39%), and exposure keratopathy (10, 28, 31, 33, 35, 36, 43, 47–49, 62). In addition, the corneal sensation may only return to a normal level after 12 months post-PKP and DALK, and in some cases never fully recover, which renders the cornea more susceptible to epithelial breakdown and infection (82). It is also worth noting that bullous keratopathy secondary to graft failure, following any type of keratoplasty, serves as another important risk factor (6–61%) for PKIK (Figures 1C–F).

### Cornea Preservation Method

Currently, two main methods are used to store and preserve donor corneas in the eye banks, including organ culture and hypothermia (83). Organ culture involves suspending corneal tissues in cell culture medium (most commonly Eagle's minimum essential medium) with foetal bovine serum. Antibiotics and antifungals are added to prevent growth of microorganisms (83). Additionally, frequent testing of the suspension medium for microbial growth is conducted to ensure the sterility before transplantation. This method is able to preserve corneas for up to 4 weeks at 28–37°C (83). On the other hand, the hypothermia method utilises storage medium such as Optisol-GS (which contains dextran and chondroitin sulphate) to prevent corneal oedema and is able to preserve corneas for 7–14 days at a temperature of 2–8°C (83). While presenting a clear advantage of its technical simplicity, the lower storage temperature and shorter storage duration may reduce the chance of microbial detection before transplantation, thereby increasing the risk of PKIK (84, 85).

Hypothermic storage has been shown to have a higher positive rim culture rate (9.8%) compared to organ culture (1.3%) (84). Similarly, a Spanish study observed a 3.2% positive microbiological culture among 1,369 donor corneoscleral rims and found that 61.8% were related to corneas stored in hypothermia (86). Notably, the preferred hypothermic storage medium used in Europe and US contains only antibiotics (e.g., gentamicin) but without an antifungal agent as seen in the organ culture medium (29, 85). This may explain the higher rates of PKIK due to *Candida spp*. following EK utilising hypothermic-stored corneas at European and US centres (29, 85). It is also worth noting that subsequent postoperative fungal infection is seen in 7% of the corneas with positive donor rim fungal culture (87). In view of these issues, addition of antifungal agent into the hypothermic storage medium has been proposed (88, 89). However, further investigations into the efficacy, safety and choice of antifungal agent are required as the microbiological profiles may be highly varied across different regions and antifungal agent may cause significant toxicity to endothelial cells (88).

## Causative Microorganisms

### Bacteria

Bacteria form the largest cohort of microorganisms responsible for PKIK worldwide. Although both Gramme-positive and Gramme-negative bacteria are implicated, the literature consistently highlights Gramme-positive bacteria as the most common type of organism, with up to 82.8% reported in some studies (12, 25, 26, 32–36, 39, 41–43, 45, 47, 48, 50). These Gramme-positive bacteria, which constitute the ocular surface commensals, include *Staphylococcus aureus*, closely followed by *Streptococcus pneumoniae* and *coagulase-negative Staphylococcus* such as *Staphylococcus epidermidis* (10, 12, 25–29, 32–36, 39, 41–43, 45, 47–50). Of interest, this pattern has remained the same over the last four decades. However, variable and non-stringent culture protocols across different regions could considerably impact the range of organisms reported (90).

### Fungi

The most common fungus associated with PKIK, particularly EK, are from the *Candida* species, with the majority caused by either *Candida albicans* or *Candida parapsilosis* (10, 25, 29, 33, 34, 43, 47, 48, 50, 89, 91–94). The other less commonly reported fungi implicated in PKIK include *Fusarium spp*. and *Aspergillus spp*., as well as rare organisms such as *Cryptococcus spp*. and *Arthrographis spp*. (28, 34, 49, 50, 95, 96).

### Viruses

Herpes simplex keratitis (HSK) represents an important cause for PKIK. Although accounting for <7% of microbial causes of PKIK (29, 35), the incidence calculated by a Dutch study of 2,112 patients is 1.2 per 1,000 person-years (97). Comparatively, a Chinese study of 1,443 patients found the incidence to be 1.2% (98). Post-keratoplasty HSK can present as classic dendritic keratitis, geographic ulcer, or non-healing epithelial defect (97). Interestingly, HSK may develop following keratoplasty despite no previous diagnosis, with the majority presenting within 2 years of transplantation (97). This is likely to be attributable to the high seropositive rate within the population despite being asymptomatic and the suppression of local immunity with corticosteroids use (98, 99). As such, HSV should be considered as a diagnosis in cases of non-resolving epithelial defects following keratoplasty. Cytomegalovirus (CMV) infection is another important cause of graft infection, rejection and failure following PKP and EK, though it is most commonly reported in East Asia (100, 101). A recent UK study failed to identify the presence of CMV in any of the 92 cases of failed corneal graft tissues, suggesting that CMV may be a region-specific risk factor for graft infection and failure (102).

### Others

Apart from recurrences following therapeutic keratoplasty, other types of IK, secondary to *Acanthamoeba* and acid-fast bacillus (e.g., non-tuberculous *Mycobacterium*), have rarely been reported after keratoplasty (12, 33, 103).

## Diagnosis

### Microscopy, Culture, and Sensitivity

The diagnostic approach for PKIK is similar to “standard” IK, unless the PKIK is related to IIK. Corneal scraping for microscopy, culture and sensitivity testing represents the gold standard for diagnosing IK, though the culture yield varies between 24 and 77% (1, 2, 104, 105). To maximise collection of microorganisms for culture, the corneal scrape should be taken at the ulcer base or leading edge. Microscopic examination with appropriate staining (e.g., Gramme stain, Giemsa stain, potassium hydroxide with calcofluor white) serves as a more rapid diagnostic method of IK (106). Various agars are used for culturing the causative microorganisms, including blood/chocolate agar (for bacteria), Sabouraud dextrose agar (for fungi), and non-nutrient agar with *Escherichia coli* overlay (for *Acanthamoeba*).

In suture-related PKIK cases, infected corneal sutures should also be sent for microbiological culture as they may provide additional information. Adler et al. (107) evaluated the presence of microbial growth and biofilm formation amongst corneal sutures removed following astigmatic correction (quiescent), loosening or breakage (exposed), or infection. Biofilms are composed of extracellular matrix secreted by microorganisms and are usually resistant to conventional antimicrobial treatment (108, 109). They have been shown to form on biotic and abiotic surfaces, including sutures (107, 110). In their study, the infection group demonstrated a culture yield of 60% based on corneal sutures, underlining corneal suture material (when infected) as a useful source for obtaining microbiological diagnosis. In addition, higher biofilm scores (on scanning electron microscopy) were observed in the infection and exposed groups, highlighting the importance of early removal of sutures to prevent suture-related PKIK.

### *In vivo* Confocal Microscopy

*In vivo* confocal microscopy (IVCM) is a non-invasive diagnostic investigation allowing visualisation of the cornea at high resolutions of 1–2 μm by limiting scattered light and focusing the observation system to a single point (111). As such, it offers a useful tool for determining fungal keratitis and AK (but not bacterial keratitis). IVCM has the benefit of providing rapid diagnoses but the diagnostic accuracy is reliant on the operator's experience (112). Compared to culture, IVCM is able to detect fungal filaments with a sensitivity of 85.7–94% and a specificity of 78–81.4% (113, 114). Similarly for AK, the sensitivity and specificity are 88.2–100 and 98.2–100%, respectively (113, 115, 116). Additionally, IVCM lends itself as an important investigation for determining the causes of IIK where access for corneal sampling is limited (22, 117). Recently, artificial intelligence (AI)-assisted diagnosis based on IVCM images has been shown to reliably diagnose fungal keratitis (118, 119).

### Anterior Segment Optical Coherence Tomography

**Figure 2:**
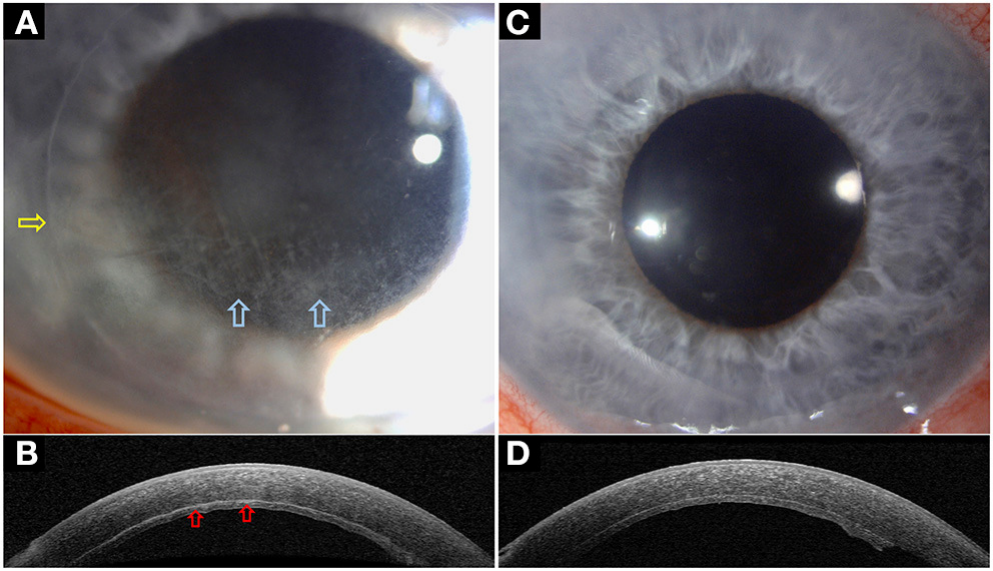
A case of interface infectious keratitis (IIK) following Descemet stripping automated endothelial keratoplasty (DSAEK). **(A,B)** Slit-lamp photography demonstrating an inflamed right eye with diffused stromal haze in a crisscross pattern at the graft-host interface (blue arrows), consistent with a diagnosis of IIK. The edge of the DSAEK graft is visible (yellow arrow). The hyper-reflective changes at the graft-host interface (red arrows) are clearly delineated on anterior segment optical coherence tomography (AS-OCT) highlighting the value of AS-OCT in facilitating the assessment of infectious, keratitis. **(C,D)** Slit-lamp photography demonstrating a complete resolution of the IIK following intensive topical anti-fungal treatment, evidenced by the disappearance of the stromal haze on slit-lamp photograph and the hyper-reflective changes at the graft-host interface on AS-OCT. **(A)** is reproduced from Ting et al. (22) with permission.

AS-OCT utilises low-coherence interferometry to provide high-resolution, cross-sectional imaging of the cornea (120). As AS-OCT can provide a quantitative and objective measurement of the infection, it can be used to observe characteristic patterns of IK, determine the depth and extent of IK, and monitor the progression of IK and treatment response, especially in deep-seated infection or IIK (Figure 2) (121–124). In addition, it is of particular importance in fungal keratitis as fungi have a propensity for deep-seated infection (which is more difficult to visualise on slit-lamp examination/photography) and a prolonged clinical course (125, 126).

## Treatment

**Figure 3:**
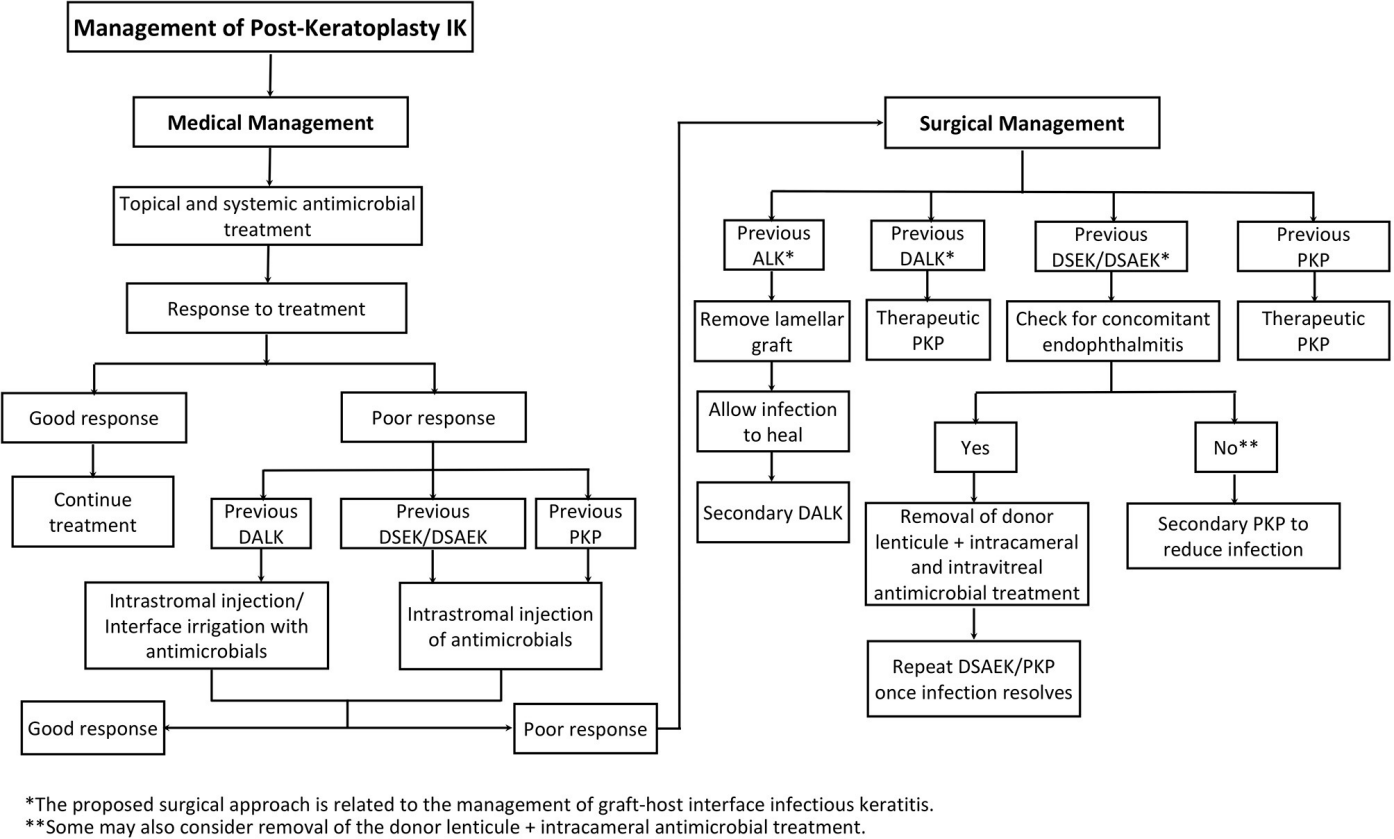
A proposed systematic treatment algorithm of post-keratoplasty infectious keratitis (PKIK). *The proposed surgical approach is related to the management of graft-host interface infectious keratitis. **Some may also consider removal of the donor lenticule + intracameral antimicrobial treatment.

The treatment strategy is guided by a number of factors, including the type of microorganism, severity, location, type of keratoplasty, and clinicians' experience and preference. A proposed systematic treatment algorithm of PKIK is illustrated in Figure 3.

### Medical Treatment

Empirical broad-spectrum antimicrobial agents are the mainstay of treatment for PKIK following both PKP and LK. Commonly used topical antibiotics include fluoroquinolones and fortified cephalosporins and aminoglycosides. Depending on the type of fungal infection (filamentous vs. yeast), antifungal treatment such as natamycin, amphotericin B, and voriconazole are commonly administered. Early concurrent systemic antifungal treatment is often initiated in severe cases (127, 128). Subsequent medical treatment is then tailored to the clinical progress and microbiological results. Akova et al. (36) reported successful medical management in 43% eyes of the 21 eyes with IK following PKP. Vajpayee et al. (62) observed a success rate of 74% in resolving PKIK with medical management alone. In the event where the use of topical steroids is contraindicated in grafted patients (e.g., infection or steroid responder), topical ciclosporin may serve as a useful substitute (129, 130).

**Figure 4:**
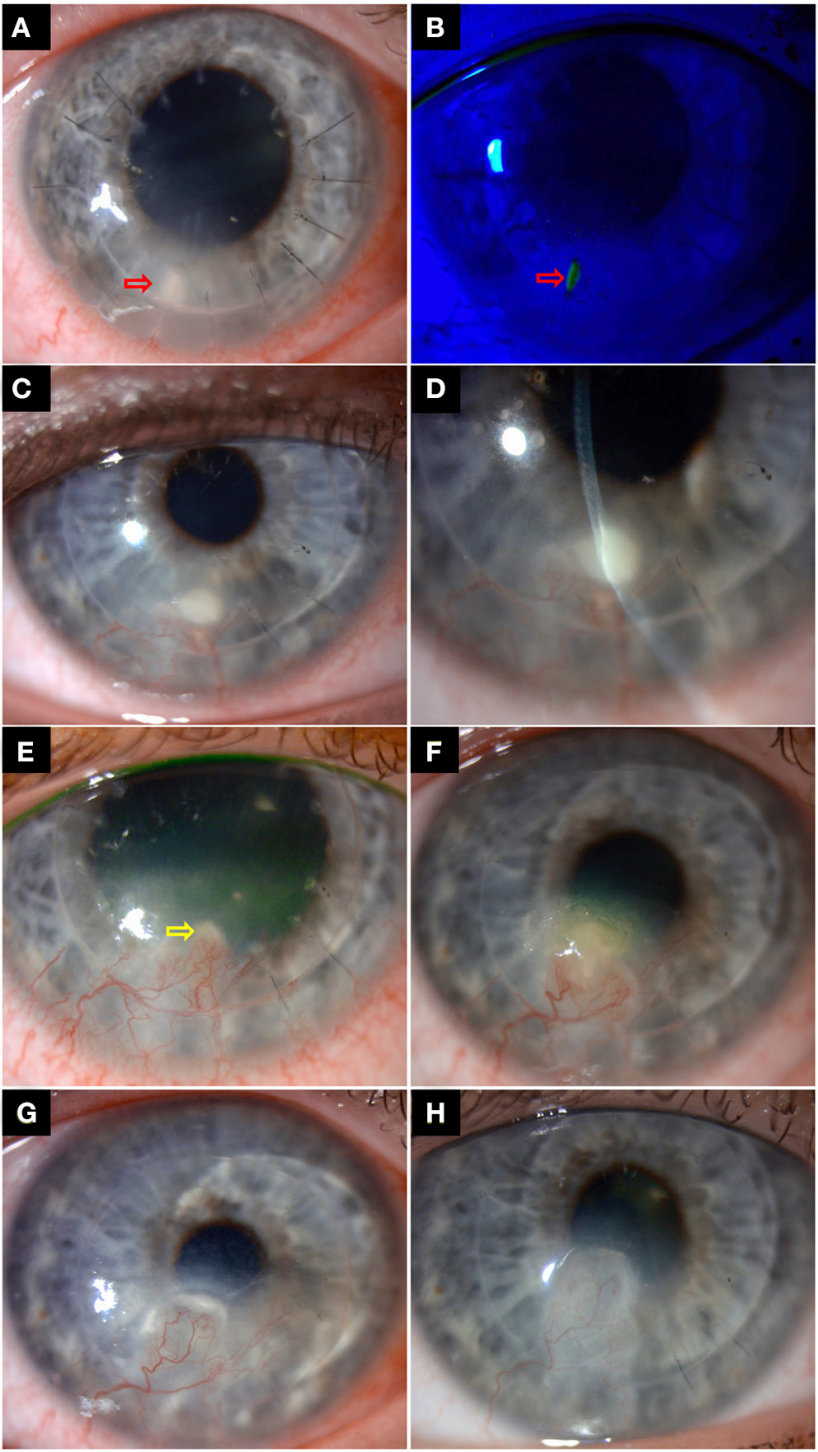
A case of right recurrent interface infectious keratitis (IIK) after deep anterior lamellar keratoplasty (using manual dissection technique) for keratoconus. **(A,B)** Slit-lamp photography in Aug 2018 demonstrating a suture-related infection, with a mid-stromal infiltrate and a small overlying epithelial defect along the suture track at 7 o'clock (red arrows), with surrounding stromal oedema/folds. The infected broken suture was removed, and the infection was successfully resolved with topical antibiotic treatment. **(C,D)** A year later, slit-lamp photography showing a recurrent mid-to-deep stromal infiltrate (involving the graft-host interface) at the same site with inferior corneal graft vascularization, suggesting an atypical presentation of IIK. The recurrence was likely due to a “reactivation” of the previously treated infective nidus at the graft-host interface. **(E)** Improvement of the superficial infection was observed after two weeks of intensive antibiotic treatment. The residual IIK (yellow arrow) was resolved after a further 3 weeks of topical antibiotic treatment. **(F)** Further recurrence of infection was again observed in October 2020. Note the gradual migration of the infection towards the visual axis along the graft-host interface, compared to the previous years. **(G)** The patient was treated for a mixed bacterial/fungal infection with intensive topical antibiotic and antifungal treatment, but only a partial response was observed. A course of repeated intrastromal injections of voriconazole 0.1% (0.1 ml) was subsequently given every weekly for 4 weeks. **(H)** Complete resolution of infection was achieved, with a residual scar.

IIK frequently poses a significant therapeutic challenge due to the deep location of the infective nidus and entrapment of organisms in the interface between host and donor tissues (Figure 4) (21, 22, 91). Topical medications often have limited penetration to the deep cornea and fail to reach a therapeutic concentration at the site of infection. Epithelial debridement may improve drug penetration. Although uncommon, the interface infection may be heralded by an ocular surface infection or may extend from the interface to the ocular surface, both of which allow for scraping for microbiological culture and better penetration of topical treatment (22, 131). However, medical treatment alone has been shown to achieve successful eradication of infection in only 13.3–24.2% cases of IIK, with high proportion requiring surgical interventions (21, 91, 117, 132).

Intrastromal injections or interface irrigation with antimicrobial agents may be used when the infection is not responding to topical treatment, particularly in deep-seated infection and IIK (Figure 4) (127, 132–134). Kalaiselvi et al. (135) demonstrated that intrastromal voriconazole injection was able to resolve 72% of deep recalcitrant fungal keratitis that did not respond to topical natamycin and natamycin drops. Tu and Hou (134) reported successful resolution of two cases of post-DSAEK fungal IIK with repeated intrastromal antifungal injection, obviating the need for PKP. However, it is important to bear in mind that excessive injection of treatment extending into the interface may weaken the graft-host attachment and risk graft detachment and endophthalmitis (91, 127, 134). In addition, a recent review showed that only 10% of the reported cases of post-DSAEK IIK resolved without any surgical intervention, highlighting the therapeutic challenge of this clinical entity (91). Interface irrigation with antibacterial agents such as vancomycin (5%) has reportedly been effective in clearing DALK-related IIK (132). Use of antifungal agents such as amphotericin B (0.15–0.5%), voriconazole (1.0–5.0%), and fluconazole (5%) have also been described (91, 133, 136, 137). Apart from treatment, Wessel et al. (133) have suggested using the irrigation fluid obtained after interface wash for microbiological investigations. Although rare, risk of Descemet membrane (DM) perforation needs to be kept in mind in these cases (128, 138, 139).

### Surgical Treatment

The choice of surgical treatment of PKIK is dependent on the extent of infection and types of primary keratoplasty. In cases of IK following PKP, therapeutic PKP is needed in large ulcers not responding to medical treatment whereas optical PKP can be performed at a later stage to remove significant IK scarring and improve vision (140). Studies have reported that an emergency TPK was required in ~15–20% cases of severe PKIK and some (up to 10%) may even require evisceration if it progresses to endophthalmitis (36, 62).

In cases with ALK, the choice of surgical treatment depends on the primary procedure. In cases where adequate host stroma is left behind in the primary procedure, such as in manual or automated ALK, the lamellar graft can be removed and the surface allowed to re-epithelialise whilst on antimicrobial treatment (141, 142). This helps reduce the microbial load and facilitate the corneal healing (143). Once the infection has healed, secondary DALK may be considered (144–146). In cases where the primary procedure was DALK, the interface is more likely to provide a potential space for sequestration of infection resulting in recurrences later (147). Although clear grafts have been achieved in repeat DALK procedures following IIK post-DALK (136, 148), recurrences of infections have been reported with cases then requiring PKP (147, 149). Emergency therapeutic PKP may be required in cases with non-responding infiltrates and impending/actual perforations (139, 147).

In cases of DSAEK, removal of donor lenticule may lead to intraocular spread of infection resulting in endophthalmitis (21). However, in the presence of concomitant endophthalmitis, removal of the donor lenticule helps by reducing the microbial load and aids faster resolution (150). As such, removal of donor lenticule is not recommended unless there is concomitant endophthalmitis. A repeat DSAEK can be considered once complete resolution of infection is achieved and if host cornea is clear (151). In medically refractory IIK post-DSAEK, an early excisional PKP (including the removal of the infected DSAEK) is advisable as it helps remove the interface infection and prevents intraocular spread of infection and subsequent endophthalmitis (152). Few cases of IIK have been reported after DMEK. Thompson et al. (153) reported a case of fungal keratitis and endophthalmitis post-DMEK. The authors removed the DMEK graft and administered intravitreal antifungal agents every alternate day until the infection resolved. DSAEK was then performed as a secondary procedure, which achieved a final best-corrected-visual-acuity (BCVA) of 6/18. Another case of post-DMEK interface fungal keratitis with endophthalmitis was reported by Doshi et al. (154). Initial conservative treatment with intracameral and intravitreal antifungals did not result in improvement. The patient was then subjected to pars plana vitrectomy and 3 mm of central plaque was removed from DMEK graft using a vitrector. Oral treatment with posaconazole was started and complete eradication of infection was observed at 2-month follow-up.

In the recent years, there has been an increasing popularity in the use of therapeutic corneal cross-linking (PACK-CXL) for treating bacterial and fungal keratitis, particularly in recalcitrant cases (155–157). Mikropoulos et al. (158) described an innovative use of PACK-CXL in managing a case of PKIK secondary to multidrug resistant fungal keratitis. PACK-CXL was applied to the infected graft and the affected corneoscleral rim intraoperatively followed by a same-day therapeutic keratoplasty. The graft remained free of infection during the 9-month follow-up. However, larger case series are required to examine the efficacy and safety of such approach.

## Outcome

Following treatment of PKIK, a clear graft was seen in 23–81% of eyes (10, 26, 28, 31–33, 36, 42, 43, 47–50). However, regrafts were performed in 4.5–53% of cases (25, 33, 34, 36, 42, 47, 49). The visual outcome varied among studies, with only 20.6–56% of eyes achieving a final BCVA of ≥6/60 (12, 28, 48). Wagoner et al. (12) found that whilst 59.8% had ambulatory vision (counting fingers or better), only 7.8% had a BCVA of ≥6/12 after recovering from PKIK. Interestingly, extreme of age (either <12 or >60 years) was a poor prognostic factors for visual outcome (12). In a study with patients who received therapeutic PKP to treat medically-uncontrolled IK, a clear graft was sustained in 47.4% eyes at 2 years post-graft, with a mean BCVA of 1.8 logMAR (56). The authors proposed that these relatively poor outcomes were likely due to a combination of late surgical treatment, increased virulence of microorganisms, recurrence of infection, and reduced response to antimicrobials. As such, timely detection and management of PKIK could lead to better outcomes (56).

## Complications

A number of complications have been documented in the literature following PKIK, including graft rejection, failure, and endophthalmitis requiring evisceration/enucleation (25, 33, 42, 43, 48, 96, 159). Graft rejection and/or failure was found to occur in 7.3–71.4% cases (10, 12, 25, 26, 28, 43, 45, 49). In particular, older grafts were more likely to fail following PKIK (26). Endophthalmitis occurred in 1–13% of cases, with a large proportion of cases requiring either evisceration (75–100%) or regraft for visual rehabilitation (25%) (25, 28, 33–35, 49). Chen et al. (11) demonstrated that cause of death secondary to infection, high risk indication (i.e., infection, injury, and ulcerative keratitis), and therapeutic grafts increased the risk of endophthalmitis following penetrating keratoplasty. In addition, the time to onset of endophthalmitis may provide a useful clue to the causative organisms as bacterial infection was shown to occur significantly earlier than fungal infection (a median time of 2.5 vs. 33 days post-keratoplasty) (29). Additional complications of PKIK include corneal perforation (4.9–35%), infectious crystalline keratopathy (6%), orbital cellulitis (1%), corneal scarring (17–36%), persistent epithelial defect (39%), corneal neovascularisation (15%), wound dehiscence (11.9–35%), and phthisis bulbi (9%) (25, 26, 28, 32–35, 39, 42, 47, 49, 50, 159).

## Conclusion

PKIK is a clinical entity that often poses significant diagnostic and therapeutic challenges. It carries a high risk of serious complications such as graft rejection and failure, and less commonly endophthalmitis. PKIK after PKP and ALK is most commonly caused by ocular surface commensals, particularly Gramme-positive bacteria, whereas PKIK after EK is usually caused by *Candida spp*. Broken or loose sutures have been consistently shown to be main risk factor of PKIK and early suture removal is advocated whenever clinically possible. With the increased adoption of EK in the recent years, it is likely that the incidence of PKIK will reduce. Optimal management of ocular surface diseases such as dry eye, blepharitis, exposure keratopathy, and neurotrophic keratopathy will help reduce the risk of PKIK following PKP and DALK. Refinement in the preservation method (e.g., addition of antifungal agent in hypothermic method) may reduce the risk of graft-transmitted infection, particularly in EK. However, further investigations into the efficacy and the choice of antifungal agent are required as the microbiological profiles may be highly varied across different regions. A stepwise treatment strategy can often be used to successfully treat PKIK, though IIK often requires surgical interventions to achieve complete resolution of the infection.

## Author Contributions

DSJT: conceptualisation and supervision. AS, RD, and DSJT: data collection, curation, literature review, and manuscript drafting. HL, MA, JM, JC, DS, and HD: critical revision of manuscript. All authors approval of the final version of manuscript, data analysis, and interpretation.

## Conflict of Interest

The authors declare that the research was conducted in the absence of any commercial or financial relationships that could be construed as a potential conflict of interest.

## References

[1] Ting DSJ Ho CS Deshmukh R Said DG Dua HS. Infectious keratitis: an update on epidemiology, causative microorganisms, risk factors, and antimicrobial resistance. Eye. (2021) 35:1084–101. 10.1038/s41433-020-01339-3 33414529 PMC8102486

[2] Ung L Bispo PJ Shanbhag SS Gilmore MS Chodosh J. The persistent dilemma of microbial keratitis: Global burden, diagnosis, and antimicrobial resistance. Surv Ophthalmol. (2019) 64:255–71. 10.1016/j.survophthal.2018.12.003 30590103 PMC7021355

[3] Flaxman SR Bourne RR Resnikoff S Ackland P Braithwaite T Cicinelli MV. Global causes of blindness and distance vision impairment 1990–2020: a systematic review and meta-analysis. Lancet Glob Health. (2017) 5:e1221–34. 10.1016/S2214-109X(17)30393-5 29032195

[4] Ting DSJ Ho CS Cairns J Elsahn A Al-Aqaba M Boswell T. 12-year analysis of incidence, microbiological profiles and *in vitro* antimicrobial susceptibility of infectious keratitis: the nottingham infectious keratitis study. Br J Ophthalmol. (2021) 105:328–33. 10.1136/bjophthalmol-2020-316128 32580955 PMC7907586

[5] Khoo P Cabrera-Aguas MP Nguyen V Lahra MM Watson SL. Microbial keratitis in Sydney, Australia: risk factors, patient outcomes, and seasonal variation. Graefes Arch Clin Exp Ophthalmol. (2020) 258:1745–55. 10.1007/s00417-020-04681-0 32358645

[6] Akpek EK Gottsch JD. Immune defense at the ocular surface. Eye. (2003) 17:949–56. 10.1038/sj.eye.6700617 14631402

[7] Mohammed I Said DG Dua HS. Human antimicrobial peptides in ocular surface defense. Prog Retin Eye Res. (2017) 61:1–22. 10.1016/j.preteyeres.2017.03.004 28587935

[8] Ng AL-K To KK-W Yuen LH Yim S-M Chan KS-K Lai JS-M. Predisposing factors, microbial characteristics, and clinical outcome of microbial keratitis in a tertiary centre in Hong Kong: a 10-year experience. J Ophthalmol. (2015) 2015:769436. 10.1155/2015/769436 26167295 PMC4488544

[9] Ting DSJ Cairns J Gopal BP Ho CS Krstic L Elsahn A. Risk factors, clinical outcomes and prognostic factors of bacterial keratitis: the nottingham infectious keratitis study. medRxiv. (2021) 2021.05.26.21257881. 10.1101/2021.05.26.21257881 PMC8385317 34458289

[10] Lin I-H Chang Y-S Tseng S-H Huang Y-H. A comparative, retrospective, observational study of the clinical and microbiological profiles of post-penetrating keratoplasty keratitis. Sci Rep. (2016) 6:32751. 10.1038/srep32751 27587283 PMC5009354

[11] Chen JY Jones MN Srinivasan S Neal TJ Armitage WJ Kaye SB. Endophthalmitis after penetrating keratoplasty. Ophthalmology. (2015) 122:25–30. 10.1016/j.ophtha.2014.07.038 25264028

[12] Wagoner MD Al-Swailem SA Sutphin JE Zimmerman MB. Bacterial keratitis after penetrating keratoplasty: incidence, microbiological profile, graft survival, and visual outcome. Ophthalmology. (2007) 114:1073–9.e2. 10.1016/j.ophtha.2006.10.015 17275089

[13] Tan DT Dart JK Holland EJ Kinoshita S. Corneal transplantation. Lancet. (2012) 379:1749–61. 10.1016/S0140-6736(12)60437-1 22559901

[14] Ting DS Sau C Srinivasan S Ramaesh K Mantry S Roberts F. Changing trends in keratoplasty in the West of Scotland: a 10-year review. Br J Ophthalmol. (2012) 96:405–8. 10.1136/bjophthalmol-2011-300244 21733923

[15] Park CY Lee JK Gore PK Lim C-Y Chuck RS. Keratoplasty in the United States: a 10-year review from 2005 through 2014. Ophthalmology. (2015) 122:2432–42. 10.1016/j.ophtha.2015.08.017 26386848

[16] Ross AR Said DG Colabelli Gisoldi RAM Nubile M El-Amin A Gabr AF. Optimizing pre-Descemet endothelial keratoplasty technique. J Cataract Refract Surg. (2020) 46:667–74. 10.1097/j.jcrs.0000000000000157 32358258

[17] Ang M Wilkins MR Mehta JS Tan D. Descemet membrane endothelial keratoplasty. Br J Ophthalmol. (2016) 100:15–21. 10.1136/bjophthalmol-2015-306837 25990654

[18] Jankowska-Szmul J Dobrowolski D Krysik K Kwas J Nejman M Wylegala E. Changes in technique and indications for keratoplasty in Poland, 1989 to 2014: an analysis of corneal transplantations performed at saint barbara hospital, Trauma center, Sosnowiec, Poland. Transplant Proc. (2016) 48:1818–23. 10.1016/j.transproceed.2016.01.056 27496499

[19] Ang M Ting DSJ Kumar A May KO Htoon HM Mehta JS. Descemet membrane endothelial keratoplasty in Asian eyes: intraoperative and postoperative complications. Cornea. (2020) 39:940–5. 10.1097/ICO.0000000000002302 32452991

[20] Deshmukh R Nair S Ting DSJ Agarwal T Beltz J Vajpayee RB. Graft detachments in endothelial keratoplasty. Br J Ophthalmol. (2021). 10.1136/bjophthalmol-2020-318092. [Epub ahead of print]. 33397659

[21] Sharma N Kaur M Titiyal JS Aldave A. Infectious keratitis after lamellar keratoplasty. Surv Ophthalmol. (2020) 66:623–43. 10.1016/j.survophthal.2020.11.001 33217327

[22] Ting DSJ Said DG Dua HS. Interface haze after descemet stripping automated endothelial keratoplasty. JAMA Ophthalmol. (2019) 137:1201–2. 10.1001/jamaophthalmol.2019.2745 31393546

[23] Ang M Mehta JS Mantoo S Tan D. Deep anterior lamellar keratoplasty to treat microsporidial stromal keratitis. Cornea. (2009) 28:832–5. 10.1097/ICO.0b013e3181930ddc 19574897

[24] Dohse N Wibbelsman TD Rapuano SB Hammersmith KM Nagra PK Rapuano CJ. Microbial keratitis and clinical outcomes following penetrating and endothelial keratoplasty. Acta Ophthalmol. (2020) 98:e895–900. 10.1111/aos.14404 32190979

[25] Griffin B Walkden A Okonkwo A Au L Brahma A Carley F. Microbial keratitis in corneal transplants: a 12-year analysis. Clin Ophthalmol. (2020) 14:3591. 10.2147/OPTH.S275067 33154618 PMC7605946

[26] Okonkwo A Siah W Hogg H Anwar H Figueiredo F. Microbial keratitis in corneal grafts: predisposing factors and outcomes. Eye. (2018) 32:775–81. 10.1038/eye.2017.310 29386617 PMC5898877

[27] Sun JP Chen WL Huang JY Hou YC Wang IJ Hu FR. Microbial keratitis after penetrating keratoplasty. Am J Ophthalmol. (2017) 178:150–6. 10.1016/j.ajo.2017.03.022 28347669

[28] Chen HC Lee CY Lin HY Ma DHK Chen PYF Hsiao C-H. Shifting trends in microbial keratitis following penetrating keratoplasty in Taiwan. Medicine. (2017) 96:e5864. 10.1097/MD.0000000000005864 28151861 PMC5293424

[29] Edelstein SL DeMatteo J Stoeger CG Macsai MS Wang CH. Report of the eye bank association of America medical review subcommittee on adverse reactions reported from 2007 to 2014. Cornea. (2016) 35:917–26. 10.1097/ICO.0000000000000869 27158807

[30] Constantinou M Jhanji V Vajpayee RB. Clinical and microbiological profile of post-penetrating keratoplasty infectious keratitis in failed and clear grafts. Am J Ophthalmol. (2013) 155:233–7.e2. 10.1016/j.ajo.2012.07.026 23111174

[31] Tavakkoli H Sugar J. Microbial keratitis following penetrating keratoplasty. Ophthalmic Surg. (1994) 25:356–60. 10.3928/1542-8877-19940601-04 8090413

[32] Leahey AB Avery RL Gottsch JD Mallette RA Stark WJ. Suture abscesses after penetrating keratoplasty. Cornea. (1993) 12:489–92. 10.1097/00003226-199311000-00005 8261779

[33] Bates A Kirkness C Ficker L Steele A Rice N. Microbial keratitis after penetrating keratoplasty. Eye. (1990) 4:74–8. 10.1038/eye.1990.8 2323481

[34] Fong LP Ormerod LD Kenyon KR Foster CS. Microbial keratitis complicating penetrating keratoplasty. Ophthalmology. (1988) 95:1269–75. 10.1016/S0161-6420(88)33036-8 3062538

[35] Al-Hazzaa SA Tabbara KF. Bacterial keratitis after penetrating keratoplasty. Ophthalmology. (1988) 95:1504–8. 10.1016/S0161-6420(88)32988-X 3062524

[36] Akova YA Onat M Koc F Nurozler A Duman S. Microbial keratitis following penetrating keratoplasty. Ophthalmic Surg Lasers. (1999) 30:449–55. 10.3928/1542-8877-19990601-07 10392732

[37] Vajpayee RB Sharma N Sinha R Agarwal T Singhvi A. Infectious keratitis following keratoplasty. Surv Ophthalmol. (2007) 52:1–12. 10.1016/j.survophthal.2006.10.001 17212987

[38] Chan C Wong T Yeong S Lim T Tan D. Penetrating keratoplasty in the Singapore national eye centre and donor cornea acquisition in the Singapore eye bank. Ann Acad Med Singap. (1997) 26:395–400. 9395797

[39] Christo CG van Rooij J Geerards AJ Remeijer L Beekhuis WH. Suture-related complications following keratoplasty: a 5-year retrospective study. Cornea. (2001) 20:816–9. 10.1097/00003226-200111000-00008 11685058

[40] Khodadoust AA Franklin RM. Transfer of bacterial infection by donor cornea in penetrating keratoplasty. Am J Ophthalmol. (1979) 87:130–2. 10.1016/0002-9394(79)90130-2 373448

[41] Tuberville AW Wood TO. Corneal ulcers in corneal transplants. Curr Eye Res. (1981) 1:479–85. 10.3109/02713688109019989 7037312

[42] Hood CT Lee BJ Jeng BH. Incidence, occurrence rate, and characteristics of suture-related corneal infections after penetrating keratoplasty. Cornea. (2011) 30:624–8. 10.1097/ICO.0b013e3182041755 21282987

[43] Rahman I Carley F Hillarby C Brahma A Tullo A. Penetrating keratoplasty: indications, outcomes, and complications. Eye. (2009) 23:1288–94. 10.1038/eye.2008.305 18949010

[44] Crawford AZ Krishnan T Ormonde SE Patel DV McGhee CN. Corneal transplantation in New Zealand 2000 to 2009. Cornea. (2018) 37:290–5. 10.1097/ICO.0000000000001481 29227340

[45] Sharma N Prakash G Titiyal JS Tandon R Vajpayee RB. Pediatric keratoplasty in India: indications and outcomes. Cornea. (2007) 26:810–3. 10.1097/ICO.0b013e318074ce2e 17667614

[46] Toriyama K Suzuki T Shiraishi A. Characteristics of infectious keratitis in old and very old patients. J Ocul Pharmacol Ther. (2018) 34:565–9. 10.1089/jop.2018.0028 30222498

[47] Harris DJ Jr Stulting RD Waring GO III Wilson LA. Late bacterial and fungal keratitis after corneal transplantation: spectrum of pathogens, graft survival, and visual prognosis. Ophthalmology. (1988) 95:1450–7. 10.1016/S0161-6420(88)33008-3 3067181

[48] Tseng SH Ling KC. Late microbial keratitis after corneal transplantation. Cornea. (1995) 14:591–4. 10.1097/00003226-199511000-00011 8575180

[49] Huang SC Wu SC Wu WC Hong HL. Microbial keratitis—a late complication of penetrating keratoplasty. Trans R Soc Trop Med Hyg. (2000) 94:315–7. 10.1016/S0035-9203(00)90338-9 10975009

[50] Sung MS Choi W You IC Yoon KC. Factors affecting treatment outcome of graft infection following penetrating keratoplasty. Korean J Ophthalmol. (2015) 29:301–8. 10.3341/kjo.2015.29.5.301 26457035 PMC4595255

[51] Al-Yousuf N Mavrikakis I Mavrikakis E Daya S. Penetrating keratoplasty: indications over a 10 year period. Br J Ophthalmol. (2004) 88:998–1001. 10.1136/bjo.2003.031948 15258012 PMC1772260

[52] Sharma B Priyadarshini S Chaurasia S Das S. Recent advances in paediatric keratoplasty. Expert Rev Ophthalmol. (2018) 13:1–10. 10.1080/17469899.2018.1429266

[53] Majander A Kivelä TT Krootila K. Indications and outcomes of keratoplasties in children during a 40-year period. Acta Ophthalmologica. (2016) 94:618–24. 10.1111/aos.13040 27061670

[54] Dana MR Moyes AL Gomes JA Rosheim KM Schaumberg DA Laibson PR. The indications for and outcome in pediatric keratoplasty. A multicenter study. Ophthalmology. (1995) 102:1129–38. 10.1016/S0161-6420(95)30900-1 9097737

[55] Hovlykke M Hjortdal J Ehlers N Nielsen K. Clinical results of 40 years of paediatric keratoplasty in a single university eye clinic. Acta Ophthalmol. (2014) 92:370–7. 10.1111/aos.12198x 23879323

[56] Moon J Yoon CH Kim MK Oh JY. The incidence and outcomes of recurrence of infection after therapeutic penetrating keratoplasty for medically-uncontrolled infectious keratitis. J Clin Med. (2020) 9:3696. 10.3390/jcm9113696 33217910 PMC7698699

[57] Zhang Q Zhao M Xu M Gu F Liu Q Chen Y. Outcomes of therapeutic keratoplasty for severe infectious keratitis in Chongqing, a 16-year experience. Infect Drug Resist. (2019) 12:2487–93. 10.2147/IDR.S204025 31496763 PMC6697658

[58] Wan S Cheng J Dong Y Xie L. Epithelial defects after penetrating keratoplasty in infectious keratitis: an analysis of characteristics and risk factors. PLoS ONE. (2018) 13:e0208163. 10.1371/journal.pone.0208163 30485371 PMC6261636

[59] Reinhart WJ Musch DC Jacobs DS Lee WB Kaufman SC Shtein RM. Deep anterior lamellar keratoplasty as an alternative to penetrating keratoplasty a report by the american academy of ophthalmology. Ophthalmology. (2011) 118:209–18. 10.1016/j.ophtha.2010.11.002 21199711

[60] Shimazaki J Iseda A Satake Y Shimazaki-Den S. Efficacy and safety of long-term corticosteroid eye drops after penetrating keratoplasty: a prospective, randomized, clinical trial. Ophthalmology. (2012) 119:668–73. 10.1016/j.ophtha.2011.10.016 22264885

[61] Sonavane A Sharma S Gangopadhyay N Bansal AK. Clinico-microbiological correlation of suture-related graft infection following penetrating keratoplasty. Am J Ophthalmol. (2003) 135:89–91. 10.1016/S0002-9394(02)01857-3 12504703

[62] Vajpayee R Boral S Dada T Murthy G Pandey R Satpathy G. Risk factors for graft infection in India: a case-control study. Br J Ophthalmol. (2002) 86:261–5. 10.1136/bjo.86.3.261 11864877 PMC1771032

[63] Dana MR Goren MB Gomes J Laibson PR Rapuano CJ Cohen EJ. Suture erosion after penetrating keratoplasty. Cornea. (1995) 14:243–8. 10.1097/00003226-199505000-00003 7600806

[64] Sullivan LJ Su C Snibson G Taylor HR. Sterile ocular inflammatory reactions to monofilament suture material. Aust N Z J Ophthalmol. (1994) 22:175–81. 10.1111/j.1442-9071.1994.tb01713.x 7818875

[65] Siganos CS Solomon A Frucht-Pery J. Microbial findings in suture erosion after penetrating keratoplasty. Ophthalmology. (1997) 104:513–6. 10.1016/S0161-6420(97)30282-6 9082282

[66] Das S Sheorey H Taylor HR Vajpayee RB. Association between cultures of contact lens and corneal scraping in contact lens–related microbial keratitis. Arch Ophthalmol. (2007) 125:1182–5. 10.1001/archopht.125.9.1182 17846356

[67] Dana MR Schaumberg DA Moyes AL Gomes JA Laibson PR Holland EJ. Outcome of penetrating keratoplasty after ocular trauma in children. Arch Ophthalmol. (1995) 113:1503–7. 10.1001/archopht.1995.01100120033003 7487616

[68] Brightbill FS. Corneal Surgery: Theory, Technique and Tissue. Elsevier Health Sciences (2009).

[69] Sharma N Sachdev R Jhanji V Titiyal JS Vajpayee RB. Therapeutic keratoplasty for microbial keratitis. Curr Opin Ophthalmol. (2010) 21:293–300. 10.1097/ICU.0b013e32833a8e23 20531191

[70] Robaei D Carnt N Minassian DC Dart JK. Therapeutic and optical keratoplasty in the management of acanthamoeba keratitis: risk factors, outcomes, and summary of the literature. Ophthalmology. (2015) 122:17–24. 10.1016/j.ophtha.2014.07.052 25262318

[71] Kitzmann AS Goins KM Sutphin JE Wagoner MD. Keratoplasty for treatment of acanthamoeba keratitis. Ophthalmology. (2009) 116:864–9. 10.1016/j.ophtha.2008.12.029 19410943

[72] Kashiwabuchi RT De Freitas D Alvarenga LS Vieira L Contarini P Sato E. Corneal graft survival after therapeutic keratoplasty for acanthamoeba keratitis. Acta ophthalmologica. (2008) 86:666–9. 10.1111/j.1600-0420.2007.01086.x 18752517

[73] Xie L Zhai H Shi W. Penetrating keratoplasty for corneal perforations in fungal keratitis. Cornea. (2007) 26:158–62. 10.1097/01.ico.0000248381.24519.0d 17251805

[74] Chatterjee S Agrawal D. Recurrence of infection in corneal grafts after therapeutic penetrating keratoplasty for microbial keratitis. Cornea. (2020) 39:39–44. 10.1097/ICO.0000000000002044 31259861

[75] Shi W Wang T Xie L Li S Gao H Liu J. Risk factors, clinical features, and outcomes of recurrent fungal keratitis after corneal transplantation. Ophthalmology. (2010) 117:890–6. 10.1016/j.ophtha.2009.10.004 20079930

[76] Chen WL Wu CY Hu FR Wang IJ. Therapeutic penetrating keratoplasty for microbial keratitis in Taiwan from 1987 to 2001. Am J Ophthalmol. (2004) 137:736–43. 10.1016/j.ajo.2003.11.010 15059714

[77] Kaye SB Baker K Bonshek R Maseruka H Grinfeld E Tullo A. Human herpesviruses in the cornea. Br J Ophthalmol. (2000) 84:563–71. 10.1136/bjo.84.6.563 10837377 PMC1723495

[78] Lomholt JA Baggesen K Ehlers N. Recurrence and rejection rates following corneal transplantation for herpes simplex keratitis. Acta Ophthalmol. (1995) 73:29–32. 10.1111/j.1600-0420.1995.tb00008.x 7627755

[79] Awan MA Roberts F Hegarty B Ramaesh K. The outcome of deep anterior lamellar keratoplasty in herpes simplex virus-related corneal scarring, complications and graft survival. Br J Ophthalmol. (2010) 94:1300–3. 10.1136/bjo.2009.169300 20554507

[80] Bhatt UK Abdul Karim MN Prydal JI Maharajan SV Fares U. Oral antivirals for preventing recurrent herpes simplex keratitis in people with corneal grafts. Cochrane Database Syst Rev. (2016) 11:Cd007824. 10.1002/14651858.CD007824.pub2 27902849 PMC6464863

[81] McDermott AM. Antimicrobial compounds in tears. Exp Eye Res. (2013) 117:53–61. 10.1016/j.exer.2013.07.014 23880529 PMC3844110

[82] Dua HS Said DG Messmer EM Rolando M Benitez-del-Castillo JM Hossain PN. Neurotrophic keratopathy. Prog Retin Eye Res. (2018) 66:107–31. 10.1016/j.preteyeres.2018.04.003 29698813

[83] Armitage WJ. Preservation of human cornea. Transfus Med Hemother. (2011) 38:143–7. 10.1159/000326632 21566714 PMC3088736

[84] Fontana L Errani PG Zerbinati A Musacchi Y Di Pede B Tassinari G. Frequency of positive donor rim cultures after penetrating keratoplasty using hypothermic and organ-cultured donor corneas. Cornea. (2007) 26:552–6. 10.1097/ICO.0b013e3180415d7e 17525650

[85] Lau N Sesé AH Augustin VA Kuit G Wilkins MR Tourtas T. Fungal infection after endothelial keratoplasty: association with hypothermic corneal storage. Br J Ophthalmol. (2019) 103:1487–90. 10.1136/bjophthalmol-2018-312709 30563913

[86] Sabater-Cruz N Otero N Dotti-Boada M Ríos J Gris O Güell JL. Eye bank and theatre factors for positive microbiological culture of corneoscleral rim and cornea storage medium in the real-world. Eye. (2021). 10.1038/s41433-020-01342-8. [Epub ahead of print]. 33469128 PMC8526809

[87] Mian SI Aldave AJ Tu EY Ayres BD Jeng BH Macsai MS. Incidence and outcomes of positive donor rim cultures and infections in the cornea preservation time study. Cornea. (2018) 37:1102. 10.1097/ICO.0000000000001654 29912040 PMC6081243

[88] Layer N Cevallos V Maxwell AJ Hoover C Keenan JD Jeng BH. Efficacy and safety of antifungal additives in optisol-GS corneal storage medium. JAMA Ophthalmol. (2014) 132:832–7. 10.1001/jamaophthalmol.2014.397 24789459

[89] Brothers KM Shanks RMQ Hurlbert S Kowalski RP Tu EY. Association between fungal contamination and eye bank-prepared endothelial keratoplasty tissue: temperature-dependent risk factors and antifungal supplementation of optisol-gentamicin and streptomycin. JAMA Ophthalmol. (2017) 135:1184–90. 10.1001/jamaophthalmol.2017.3797 28973097 PMC5710398

[90] Ung L Wang Y Vangel M Davies EC Gardiner M Bispo PJM. Validation of a comprehensive clinical algorithm for the assessment and treatment of microbial keratitis. Am J Ophthalmol. (2020) 214:97–109. 10.1016/j.ajo.2019.12.019 31899203

[91] Fontana L Moramarco A Mandarà E Russello G Iovieno A. Interface infectious keratitis after anterior and posterior lamellar keratoplasty. Clinical features and treatment strategies. A review. Br J Ophthalmol. (2019) 103:307–14. 10.1136/bjophthalmol-2018-312938 30355718 PMC6579547

[92] Nahum Y Leon P Ricci-Filipovic BA Camposampiero D Ponzin D Busin M. Asymptomatic infection in decompensated full-thickness corneal grafts referred for repeat penetrating keratoplasty. Cornea. (2017) 36:431–3. 10.1097/ICO.0000000000001121 28129295

[93] Vislisel JM Goins KM Wagoner MD Schmidt GA Aldrich BT Skeie JM. Incidence and outcomes of positive donor corneoscleral rim fungal cultures after keratoplasty. Ophthalmology. (2017) 124:36–42. 10.1016/j.ophtha.2016.09.017 27817919

[94] Tsui E Fogel E Hansen K Talbot EA Tammer R Fogel J. Candida interface infections after descemet stripping automated endothelial keratoplasty. Cornea. (2016) 35:456–64. 10.1097/ICO.0000000000000778 26890665

[95] Ting DSJ Bignardi G Koerner R Irion LD Johnson E Morgan SJ. Polymicrobial keratitis with cryptococcus curvatus, candida parapsilosis, and stenotrophomonas maltophilia after penetrating keratoplasty: a rare case report with literature review. Eye Contact Lens. (2019) 45:e5–10. 10.1097/ICL.0000000000000517 29944507

[96] Ting DSJ McKenna M Sadiq SN Martin J Mudhar HS Meeney A. Arthrographis kalrae keratitis complicated by endophthalmitis: a case report with literature review. Eye Contact Lens. (2020) 46:e59–e65. 10.1097/ICL.0000000000000713 32443014

[97] Remeijer L Doornenbal P Geerards AJ Rijneveld WA Beekhuis WH. Newly acquired herpes simplex virus keratitis after penetrating keratoplasty. Ophthalmology. (1997) 104:648–52. 10.1016/S0161-6420(97)30257-7 9111258

[98] Qi X Wang M Li X Jia Y Li S Shi W. Characteristics of new onset herpes simplex keratitis after keratoplasty. J Ophthalmol. (2018) 2018:4351460. 10.1155/2018/4351460 30425853 PMC6217905

[99] Liesegang TJ. Herpes simplex virus epidemiology and ocular importance. Cornea. (2001) 20:1–13. 10.1097/00003226-200101000-00001 11188989

[100] Chan AS Mehta JS Al Jajeh I Iqbal J Anshu A Tan DT. Histological features of cytomegalovirus-related corneal graft infections, its associated features and clinical significance. Br J Ophthalmol. (2016) 100:601–6. 10.1136/bjophthalmol-2015-307390 26294107

[101] Anshu A Chee S-P Mehta JS Tan DT. Cytomegalovirus endotheliitis in descemet's stripping endothelial keratoplasty. Ophthalmology. (2009) 116:624–30. 10.1016/j.ophtha.2008.10.031 19195708

[102] da Costa Paula CA Gore DM Shah K Kuit G Angunawela RI Barnett JP. Cytomegalovirus infection is not a major cause of corneal graft failure in the United Kingdom. Eye. (2019) 33:833–7. 10.1038/s41433-018-0331-9 30622288 PMC6707390

[103] Moorthy RS Valluri S Rao NA. Nontuberculous mycobacterial ocular and adnexal infections. Surv Ophthalmol. (2012) 57:202–35. 10.1016/j.survophthal.2011.10.006 22516536

[104] Kaliamurthy J Kalavathy CM Parmar P Nelson Jesudasan CA Thomas PA. Spectrum of bacterial keratitis at a tertiary eye care centre in India. Biomed Res Int. (2013) 2013:181564. 10.1155/2013/181564 24066286 PMC3770006

[105] Ting DSJ Settle C Morgan SJ Baylis O Ghosh S. A 10-year analysis of microbiological profiles of microbial keratitis: the North East England study. Eye. (2018) 32:1416–7. 10.1038/s41433-018-0085-4 29610521 PMC6085375

[106] Sharma S. Diagnosis of infectious diseases of the eye. Eye. (2012) 26:177–84. 10.1038/eye.2011.275 22094299 PMC3272189

[107] Adler E Miller D Rock O Spierer O Forster R. Microbiology and biofilm of corneal sutures. Br J Ophthalmol. (2018) 102:1602–6. 10.1136/bjophthalmol-2018-312133 30100555

[108] Flemming HC Wingender J Szewzyk U Steinberg P Rice SA Kjelleberg S. Biofilms: an emergent form of bacterial life. Nat Rev Microbiol. (2016) 14:563–75. 10.1038/nrmicro.2016.94 27510863

[109] Strugeon E Tilloy V Ploy MC Da Re S. The stringent response promotes antibiotic resistance dissemination by regulating integron integrase expression in biofilms. mBio. (2016) 7:e00868–16. 10.1128/mBio.00868-16 27531906 PMC4992968

[110] Elder MJ Stapleton F Evans E Dart JK. Biofilm-related infections in ophthalmology. Eye. (1995) 9 (Pt. 1):102–9. 10.1038/eye.1995.16 7713236

[111] Jalbert I Stapleton F Papas E Sweeney D Coroneo M. In vivo confocal microscopy of the human cornea. Br J Ophthalmol. (2003) 87:225–36. 10.1136/bjo.87.2.225 12543757 PMC1771516

[112] Kumar RL Cruzat A Hamrah P. Current state of *in vivo* confocal microscopy in management of microbial keratitis. Semin Ophthalmol. (2010) 25:166–70. 10.3109/08820538.2010.518516 21090995 PMC3157328

[113] Chidambaram JD Prajna NV Larke NL Palepu S Lanjewar S Shah M. Prospective study of the diagnostic accuracy of the in vivo laser scanning confocal microscope for severe microbial keratitis. Ophthalmology. (2016) 123:2285–93. 10.1016/j.ophtha.2016.07.009 27538797 PMC5081072

[114] Kanavi MR Javadi M Yazdani S Mirdehghanm S. Sensitivity and specificity of confocal scan in the diagnosis of infectious keratitis. Cornea. (2007) 26:782–6. 10.1097/ICO.0b013e318064582d 17667609

[115] Tu EY Joslin CE Sugar J Booton GC Shoff ME Fuerst PA. The relative value of confocal microscopy and superficial corneal scrapings in the diagnosis of acanthamoeba keratitis. Cornea. (2008) 27:764–72. 10.1097/ICO.0b013e31816f27bf 18650660

[116] Goh JW Harrison R Hau S Alexander CL Tole DM Avadhanam VS. Comparison of in vivo confocal microscopy, PCR and culture of corneal scrapes in the diagnosis of acanthamoeba keratitis. Cornea. (2018) 37:480–5. 10.1097/ICO.0000000000001497 29256983

[117] Gao Y Li C Bu P Zhang L Bouchard CS. Infectious interface keratitis (IIK) following lamellar keratoplasty: a literature review. Ocul Surf. (2019) 17:635–43. 10.1016/j.jtos.2019.08.001 31415815

[118] Ting DSJ Foo VH Yang LWY Sia JT Ang M Lin H. Artificial intelligence for anterior segment diseases: emerging applications in ophthalmology. Br J Ophthalmol. (2021) 105:158–68. 10.1136/bjophthalmol-2019-315651 32532762

[119] Lv J Zhang K Chen Q Chen Q Huang W Cui L. Deep learning-based automated diagnosis of fungal keratitis with in vivo confocal microscopy images. Ann Transl Med. (2020) 8:706. 10.21037/atm.2020.03.134 32617326 PMC7327373

[120] Ang M Baskaran M Werkmeister RM Chua J Schmidl D Aranha Dos Santos V. Anterior segment optical coherence tomography. Prog Retin Eye Res. (2018) 66:132–56. 10.1016/j.preteyeres.2018.04.002 29635068

[121] Sharma N Singhal D Maharana PK Agarwal T Sinha R Satpathy G. Spectral domain anterior segment optical coherence tomography in fungal keratitis. Cornea. (2018) 37:1388–94. 10.1097/ICO.0000000000001715 30095493

[122] Konstantopoulos A Kuo J Anderson D Hossain P. Assessment of the use of anterior segment optical coherence tomography in microbial keratitis. Am J Ophthalmol. (2008) 146:534–42. e2. 10.1016/j.ajo.2008.05.030 18602080

[123] Soliman W Fathalla AM El-Sebaity DM Al-Hussaini AK. Spectral domain anterior segment optical coherence tomography in microbial keratitis. Graefes Arch Clin Exp Ophthalmol. (2013) 251:549–53. 10.1007/s00417-012-2086-5 22729467

[124] De Benito-Llopis L Mehta JS Angunawela RI Ang M Tan DT. Intraoperative anterior segment optical coherence tomography: a novel assessment tool during deep anterior lamellar keratoplasty. Am J Ophthalmol. (2014) 157:334–41.e3. 10.1016/j.ajo.2013.10.001 24332371

[125] Vemuganti GK Garg P Gopinathan U Naduvilath TJ John RK Buddi R. Evaluation of agent and host factors in progression of mycotic keratitis: a histologic and microbiologic study of 167 corneal buttons. Ophthalmology. (2002) 109:1538–46. 10.1016/S0161-6420(02)01088-6 12153808

[126] Gopinathan U Sharma S Garg P Rao GN. Review of epidemiological features, microbiological diagnosis and treatment outcome of microbial keratitis: experience of over a decade. Indian J Ophthalmol. (2009) 57:273–9. 10.4103/0301-4738.53051 19574694 PMC2712695

[127] Araki-Sasaki K Fukumoto A Osakabe Y Kimura H Kuroda S. The clinical characteristics of fungal keratitis in eyes after descemet's stripping and automated endothelial keratoplasty. Clin Ophthalmol. (2014) 8:1757. 10.2147/OPTH.S67326 25228792 PMC4164285

[128] Bahadir AE Bozkurt TK Kutan SA Yanyali CA Acar S. Candida interface keratitis following deep anterior lamellar keratoplasty. Int Ophthalmol. (2012) 32:383–6. 10.1007/s10792-012-9545-1 22450560

[129] Perry HD Doshi SJ Donnenfeld ED Bai GS. Topical cyclosporin A in the management of therapeutic keratoplasty for mycotic keratitis. Cornea. (2002) 21:161–3. 10.1097/00003226-200203000-00006 11862086

[130] Deshmukh R Ting DSJ Elsahn A Mohammed I Said DG Dua HS. Real-world experience of using ciclosporin-A 0.1% (Ikervis) in the management of ocular surface inflammatory diseases. Br J Ophthalmol. (2021). 10.1136/bjophthalmol-2020-317907. [Epub ahead of print]. 33687999 PMC9340021

[131] Kitzmann AS Wagoner MD Syed NA Goins KM. Donor-related candida keratitis after descemet stripping automated endothelial keratoplasty. Cornea. (2009) 28:825–8. 10.1097/ICO.0b013e31819140c4 19574899

[132] Sharma N Gupta V Vanathi M Agarwal T Vajpayee RB Satpathy G. Microbial keratitis following lamellar keratoplasty. Cornea. (2004) 23:472–8. 10.1097/01.ico.0000116525.57227.59 15220732

[133] Wessel JM Bachmann BO Meiller R Kruse FE. Fungal interface keratitis by candida orthopsilosis following deep anterior lamellar keratoplasty. Case Rep. (2013) 2013:bcr2012008361. 10.1136/bcr-2012-008361 23349184 PMC3604172

[134] Tu EY Hou J. Intrastromal antifungal injection with secondary lamellar interface infusion for late-onset infectious keratitis after DSAEK. Cornea. (2014) 33:990–3. 10.1097/ICO.0000000000000192 25055150

[135] Kalaiselvi G Narayana S Krishnan T Sengupta S. Intrastromal voriconazole for deep recalcitrant fungal keratitis: a case series. Br J Ophthalmol. (2015) 99:195–8. 10.1136/bjophthalmol-2014-305412 25185253

[136] Naik M Shahbaaz M Sheth J Sunderamoorthy S. Alternaria keratitis after deep anterior lamellar keratoplasty. Middle East Afr J Ophthalmol. (2014) 21:92–4. 10.4103/0974-9233.124121 24669155 PMC3959051

[137] Le Q Wu D Li Y Ji J Cai R Xu J. Early-onset candida glabrata interface keratitis after deep anterior lamellar keratoplasty. Optom Vis Sci. (2015) 92:e93–6. 10.1097/OPX.0000000000000565 25822017

[138] Sedaghat MR Hosseinpoor SS. Candida albicans interface infection after deep anterior lamellar keratoplasty. Indian J Ophthalmol. (2012) 60:328–30. 10.4103/0301-4738.98723 22824609 PMC3442475

[139] Kanavi MR Foroutan AR Kamel MR Afsar N Javadi MA. Candida interface keratitis after deep anterior lamellar keratoplasty: clinical, microbiologic, histopathologic, and confocal microscopic reports. Cornea. (2007) 26:913–6. 10.1097/ICO.0b013e3180ca9a61 17721287

[140] Ang M Mehta JS Sng CC Htoon HM Tan DT. Indications, outcomes, and risk factors for failure in tectonic keratoplasty. Ophthalmology. (2012) 119:1311–9. 10.1016/j.ophtha.2012.01.021 22541633

[141] Ang M Mehta JS Arundhati A Tan DT. Anterior lamellar keratoplasty over penetrating keratoplasty for optical, therapeutic, and tectonic indications: a case series. Am J Ophthalmol. (2009) 147:697–702.e2. 10.1016/j.ajo.2008.10.002 19058778

[142] Ang M Mohamed-Noriega K Mehta JS Tan D. Deep anterior lamellar keratoplasty: surgical techniques, challenges, and management of intraoperative complications. Int Ophthalmol Clin. (2013) 53:47–58. 10.1097/IIO.0b013e31827eb746 23470588

[143] Ang M Mehta JS. Deep anterior lamellar keratoplasty as an alternative to penetrating keratoplasty. Ophthalmology. (2011) 118:2306–7; author reply 7. 10.1016/j.ophtha.2011.07.025 22047896

[144] Lyall DA Srinivasan S Roberts F. A case of interface keratitis following anterior lamellar keratoplasty. Surv Ophthalmol. (2012) 57:551–7. 10.1016/j.survophthal.2012.01.010 22542911

[145] Bi Y-L Bock F Zhou Q Cursiefen C. Recurrent interface abscess secondary to acanthamoeba keratitis treated by deep anterior lamellar keratoplasty. Int J Ophthalmol. (2012) 5:774–5. 10.3980/j.issn.2222-3959.2012.06.22 23275916 PMC3530824

[146] Alio Del Barrio JL Bhogal M Ang M Ziaei M Robbie S Montesel A. Corneal transplantation after failed grafts: options and outcomes. Surv Ophthalmol. (2021) 66:20–40. 10.1016/j.survophthal.2020.10.003 33065176

[147] Bajracharya L Sharma B Gurung R. A case of acute postoperative keratitis after deep anterior lamellar keratoplasty by multidrug resistant klebsiella. Indian J Ophthalmol. (2015) 63:344–6. 10.4103/0301-4738.158088 26044477 PMC4463562

[148] Jafarinasab M-R Feizi S Yazdizadeh F Kanavi MR Moein H-R. Aspergillus flavus keratitis after deep anterior lamellar keratoplasty. J Ophthalmic Vis Res. (2012) 7:167–71. 23275826 PMC3520469

[149] Fontana L Parente G Di Pede B Tassinari G. Candida albicans interface infection after deep anterior lamellar keratoplasty. Cornea. (2007) 26:883–5. 10.1097/ICO.0b013e318074e475 17667630

[150] Kaiura TL Ritterband DC Koplin RS Shih C Palmiero PM Seedor JA. Endophthalmitis after descemet stripping endothelial keratoplasty with concave-oriented dislocation on slit-lamp optical coherence topography. Cornea. (2010) 29:222–4. 10.1097/ICO.0b013e3181a325c1 19770723

[151] Ang M Ho H Wong C Htoon HM Mehta JS Tan D. Endothelial keratoplasty after failed penetrating keratoplasty: an alternative to repeat penetrating keratoplasty. Am J Ophthalmol. (2014) 158:1221–7.e1. 10.1016/j.ajo.2014.08.024 25152499

[152] Lee WB Foster JB Kozarsky AM Zhang Q Grossniklaus HE. Interface fungal keratitis after endothelial keratoplasty: a clinicopathological report. Ophthalmic Surg Lasers Imaging. (2011) 42:e44–8. 10.3928/15428877-20110407-01 21485974

[153] Thompson M Carli D. First reported case of donor related candida endophthalmitis after descemet membrane endothelial keratoplasty. Open Ophthalmol J. (2017) 11:117–21. 10.2174/1874364101711010117 28761565 PMC5510561

[154] Doshi H Pabon S Price MO Feng MT Price FW Jr. Overview of systemic candida infections in hospital settings and report of candida after DMEK successfully treated with antifungals and partial graft excision. Cornea. (2018) 37:1071–4. 10.1097/ICO.0000000000001608 29634675

[155] Ting DSJ Henein C Said DG Dua HS. Photoactivated chromophore for infectious keratitis - corneal cross-linking (PACK-CXL): a systematic review and meta-analysis. Ocul Surf. (2019) 17:624–34. 10.1016/j.jtos.2019.08.006 31401338

[156] Prajna NV Radhakrishnan N Lalitha P Austin A Ray KJ Keenan JD. Cross-linking-assisted infection reduction: a randomized clinical trial evaluating the effect of adjuvant cross-linking on outcomes in fungal keratitis. Ophthalmology. (2020) 127:159–66. 10.1016/j.ophtha.2019.08.029 31619359 PMC6982573

[157] Ting DSJ Henein C Said DG Dua HS. Re: prajna et al.: cross-linking-assisted infection reduction (CLAIR): A randomized clinical trial evaluating the effect of adjuvant cross-linking on outcomes in fungal keratitis (Ophthalmology. (2020). 127:159–166). Ophthalmology. (2020) 127:e55–6. 10.1016/j.ophtha.2020.02.032 32703392

[158] Mikropoulos DG Kymionis GD Voulgari N Kaisari E Nikolakopoulos KA Katsanos A. Intraoperative photoactivated chromophore for infectious keratitis-corneal cross-linking (PACK-CXL) during penetrating keratoplasty for the management of fungal keratitis in an immunocompromised patient. Ophthalmol Ther. (2019) 8:491–5. 10.1007/s40123-019-0196-4 31278588 PMC6692791

[159] Krysik K Wroblewska-Czajka E Lyssek-Boron A Wylegala EA Dobrowolski D. Total penetrating keratoplasty: indications, therapeutic approach, and long-term follow-up. J Ophthalmol. (2018) 2018:9580292. 10.1155/2018/9580292 29850220 PMC5933013

