# Supplementary figures and images for: Post-keratoplasty Infectious Keratitis: Epidemiology, Risk Factors, Management, and Outcomes
Source: Front Med (Lausanne). 2021 Jul 7;8:707242. doi: 10.3389/fmed.2021.707242 (PMC8292647; doi:10.3389/fmed.2021.707242)

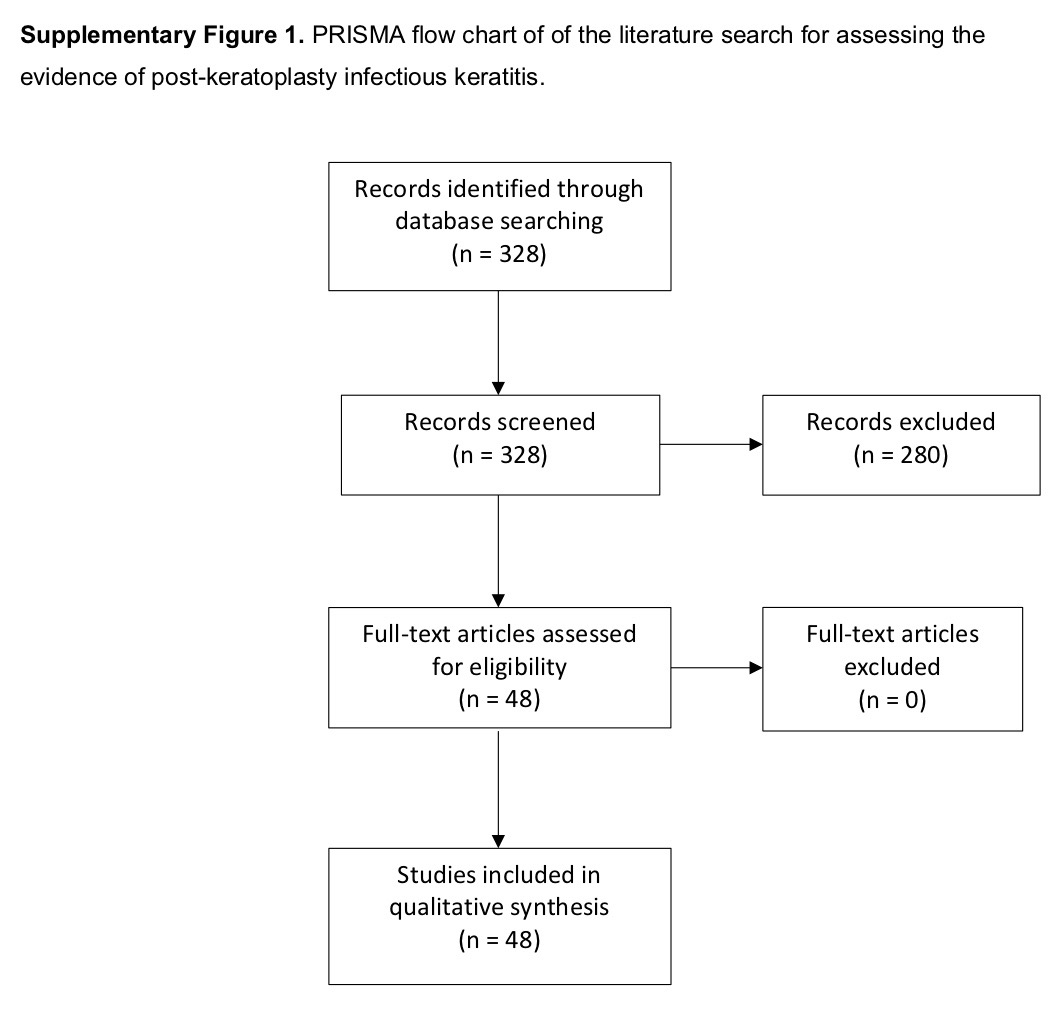

Supplement: Supplementary file 1 [file Image_1.JPEG]
